# Modification of Nanofiber Support Layer for Thin Film Composite Forward Osmosis Membranes via Layer-by-Layer Polyelectrolyte Deposition

**DOI:** 10.3390/membranes8030070

**Published:** 2018-08-25

**Authors:** Ralph Rolly Gonzales, Myoung Jun Park, Leonard Tijing, Dong Suk Han, Sherub Phuntsho, Ho Kyong Shon

**Affiliations:** 1 Centre for Technology in Water and Wastewater, University of Technology Sydney, 15 Broadway, Ultimo, New South Wales 2007, Australia; RalphRolly.Gonzales@student.uts.edu.au (R.R.G.); Myoungjun.Park@uts.edu.au (M.J.P.); Leonard.Tijing@uts.edu.au (L.T.); 2 Chemical Engineering Program, Texas A&M University at Qatar, Education City PO Box 23874, Doha, Qatar; dong_suk.han@qatar.tamu.edu

**Keywords:** membrane, forward osmosis, nanofiber, electrospinning, layered interfacial polymerization, layer-by-layer, thin film composite

## Abstract

Electrospun nanofiber-supported thin film composite membranes are among the most promising membranes for seawater desalination via forward osmosis. In this study, a high-performance electrospun polyvinylidenefluoride (PVDF) nanofiber-supported thin film composite (TFC) membrane was successfully fabricated after molecular layer-by-layer polyelectrolyte deposition. Negatively-charged electrospun polyacrylic acid (PAA) nanofibers were deposited on electrospun PVDF nanofibers to form a support layer consisted of PVDF and PAA nanofibers. This resulted to a more hydrophilic support compared to the plain PVDF nanofiber support. The PVDF-PAA nanofiber support then underwent a layer-by-layer deposition of polyethylenimine (PEI) and PAA to form a polyelectrolyte layer on the nanofiber surface prior to interfacial polymerization, which forms the selective polyamide layer of TFC membranes. The resultant PVDF-LbL TFC membrane exhibited enhanced hydrophilicity and porosity, without sacrificing mechanical strength. As a result, it showed high pure water permeability and low structural parameter values of 4.12 L m^−2^ h^−1^ bar^−1^ and 221 µm, respectively, significantly better compared to commercial FO membrane. Layer-by-layer deposition of polyelectrolyte is therefore a useful and practical modification method for fabrication of high performance nanofiber-supported TFC membrane.

## 1. Introduction

Forward osmosis (FO), a naturally-occurring physical phenomenon, is the transport of water across a selectively permeable membrane driven by the osmotic pressure difference across a membrane [1]. The membrane ideally allows only the movement of water molecules through it while rejecting the passage of solute molecules or ions. The solute concentration difference of the solutions separated by the membrane results in a difference in osmotic pressure, which drives the natural movement of water from the solution containing less amount of solute (feed solution) towards the solution containing more of the solute (draw solution). FO has been widely known as early as the 1800 s and its applications have been extensive. Not only was it used for water treatment and seawater desalination, it has also been applied to food processing, drug delivery, food preservation, and anti-microbial applications [1,2]. While FO is a naturally-occurring phenomenon, it is much less studied and developed than other water treatment and desalination processes, more specifically, reverse osmosis (RO). Among the reasons for this limitation in the study and development of FO is the lack of membranes which are designed specifically for FO [3,4,5].

The first membranes used in osmotic processes were made from plant and animal residues. An ideal selectively permeable membrane allows the solvent molecules to pass but not the solutes. Selectively permeable asymmetric cellulose acetate membrane was prepared by Sidney Loeb and Srinivasa Sourirajan in 1963, providing a breakthrough in reverse osmosis processes and membrane science [6]. However, membranes were fabricated more for application to RO rather than FO and pressure retarded osmosis (PRO). Initially, RO and nanofiltration (NF) membranes were used for FO and PRO because it was initially thought that all semi-permeable membranes can be applied for these processes. However, due to the thickness of conventional RO and NF membranes, severe internal concentration polarization (ICP) was found to occur inside the membranes. ICP present in the membranes then effectively reduces the osmotic pressure across the membranes, affecting water flux and reverse salt flux [7]. Occurrence of ICP is often associated with membrane thickness and high structural parameter; thus, membranes for FO should be designed to have high porosity and mechanical stability, while maintaining low thickness and low structural parameter value. Ever since membranes specific for FO were fabricated, FO membranes have shown better performance than RO membranes in FO processes.

Thin film composite (TFC) membranes are currently the most prepared and used membranes for osmotic process. Originally designed for pressure-driven processes such as reverse osmosis (RO) [8], TFC membranes are typically composed of an ultrathin polyamide active layer on top of a porous membrane support. The selective polyamide layer is produced via interfacial polymerization (IP) of two monomeric solutions, aqueous aromatic amine and organic multifunctional aromatic acid halide [9]. TFC membranes for forward osmosis have shown in the past to be able to achieve significantly higher water flux and salt rejection than the first generation of commercially-available symmetric cellulose acetate (CA) membrane from Hydration Technologies Innovations (HTI, Albany, OR, USA) [10]. 

Performance of FO processes is mainly affected by internal dilutive concentration polarization within the porous support layer. This is the reason why during fabrication of FO membranes, the membrane should be as thin as possible, while maintaining good strength, hydrophilicity, high porosity and low tortuosity [5]. Thin membrane thickness ensures than the structural parameter (S) is much smaller. An ideal FO membrane should have high water flux, low salt reverse, and minimal ICP [11,12]. The main goal of most recent FO membrane studies is to maintain a relatively small structural parameter, while enhancing water permeability during the formation of the membrane active layer and other post-treatment methods [13]. Addition of bulky polymers [14] and surfactants [15], nanomaterials [16], or a molecular layer-by-layer interfacial polymerization approach [17], may be done to enhance the selectivity of the active layer. However, enhancement of water permeability often enhances salt permeability as well; therefore, a balance between the two membrane parameters must be achieved.

Electrospun nanofiber membranes exhibit high porosity through its interconnected pore structure [5] and this property makes it a suitable choice for the membrane substrate for FO applications. A variety of polymeric materials can be used for electrospinning, among them, polyacrylonitrile (PAN) [18], polysulfone (PSf) [19], polyethersulfone (PES) [20], polyvinyl alcohol (PVA) [21], and polyvinylidene fluoride (PVDF) [5]. Generally, high osmotic flux and low structural parameter values were achieved for nanofiber membranes, making it a suitable method for fabrication of FO membranes. Nanofiber-supported TFC membranes applied for water-based separation processes have been the subject of various studies in the past [19,20,21,22,23,24,25,26]. While nanofiber electrospinning is a practical and non-costly method in membrane fabrication, it is still somehow limited by the electrospinning condition optimization, selection of specific materials suited for particular applications, nanofiber post-treatment [24], nanofiber strength and stability [27], membrane swelling [28], and poor adhesion of the selective polyamide layer from the nanofiber support [19]. These limitations have been addressed in various studies, yet practicality, cost, and robustness of method have yet to be fully optimized.

In this particular study, a practical integration of electrospinning, molecular layer-by-layer (LbL) approach, and interfacial polymerization was performed to improve the hydrophilicity and selectivity of the membranes, as well as the adhesion of the selective polyamide layer on the nanofiber support. The layers of the LbL approach were introduced on the electrospun PVDF nanofibers through both electrospinning and dip coating with electrolyte solutions, forming polyelectrolyte layers on the nanofiber mat, which not only improved porosity and water permeability, but also the mechanical strength and adhesion of the polyamide selective layer. These enhancements can be achieved without sacrificing the mechanical strength and stability of the membrane. This combination of nanoscale, LbL, and simplicity of IP was adapted to obtain a PVDF nanofiber-supported TFC FO membrane. The membranes were then tested for FO experiments using DI water and NaCl as the feed and draw solutions, respectively.

## 2. Materials and Methods

### 2.1. Materials

Polyvinylidene fluoride (PVDF, MW = 450,000 g mol^−1^, Kynar Powerflex®LBG, Arkema Inc., Canterbury, VIC, Australia) was used as the membrane support polymer in this study. Acetone (99.8%, Chem-Supply, Gillman, SA, Australia) and *N*,*N*-dimethylacetamide (DMAc, 99%, Sigma-Aldrich, Sydney, NSW, Australia) were used as solvents. Branched polyethylenimine (PEI, M_w_ = 750,000 g mol^−1^, Sigma-Aldrich, Saint Louise, MO, USA), poly-(acrylic acid) (PAA, M_w_ = 100,000 g mol^−1^, Sigma-Aldrich, Saint Louise, MO, USA) were used as the electrolytes. 1,3-phenylenediamine (MPD, 99%, Sigma-Aldrich, Castle Hill, NSW, Australia) and 1,3,5-benzenetricarbonyl trichloride (trimesoyl chloride, TMC, 98%, Sigma-Aldrich, Australia) were used as the precursors for IP. For the water flux test, sodium chloride (NaCl, Chem-Supply, Australia) was used as solute for the draw solution. 2-propanol (isopropyl alcohol, IPA, Sigma-Aldrich, Saint Louise, MO, USA) was used for membrane wetting. All chemicals were used as received.

### 2.2. Preparation of Nanofiber Membrane Support via Electrospinning

#### 2.2.1. Dope, Electrolyte, and Monomeric Solution Preparation

PVDF was dissolved in a 15% w/v solution with 1:1 volume ratio of acetone and DMAc as solvents. The solution was placed in magnetic stirring conditions at 60 °C for at least 12 h. PAA was dissolved in a 5 wt % solution with 0.5 M NaCl in acetone as the solvent. 0.5 wt % solutions of PEI and PAA were prepared with 0.5 M NaCl as the solvent to achieve pH of 10.6 and 3.5, respectively, to ensure the presence of the respective negative and positive charges of the solutions. 2 wt % solution of MPD and 0.15 wt % TMC solutions were also prepared with DI water and heptane as solvents.

#### 2.2.2. Electrospinning

**Figure 1:**
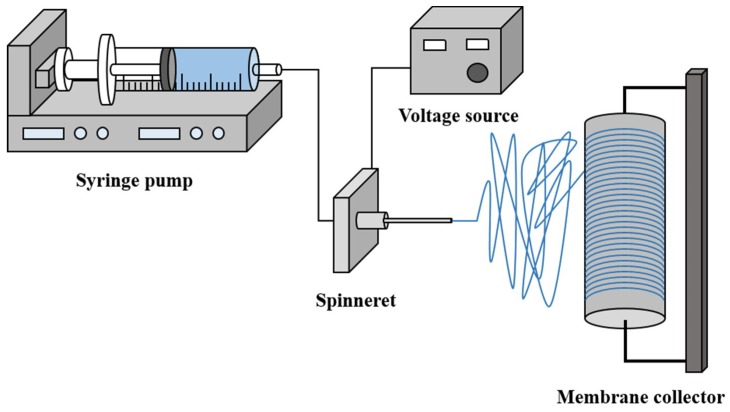
The electrospinning setup for fabrication of nanofibers.

The prepared dope solutions were charged in 10-mL syringes, placed in the electrospinning setup, as shown in Figure 1. The nanofibers were electrospun at a voltage, needle tip-to-collector distance, and solution flow rate of 22 kV, 180 mm, and 2.0 mL h^−1^, respectively. The fibers were collected onto a rotating drum collector covered with aluminium foil. The dope solutions were delivered by a syringe pump (G21, ID 0.51 mm, New Era Syringe Pump Systems, Scientific Instrument Services, Inc., Ringoes, NJ, USA) through a needle, whose inner diameter is 0.510 mm. The electrospinning process was controlled by LabView software (National Instruments, 2010 Edition, Sydney, Australia) and maintained at constant humidity (30–50%) and temperature (20–25 °C) conditions. PVDF nanofibers were first electrospun for 3 h, followed by coating with PAA nanofibers electrospun for 3 h. After electrospinning, the membranes were peeled off from the aluminium foil and placed in a temperature fan forced oven (OTWMHD24, LABEC, Sydney, Australia) to remove residual solvents. The prepared membranes were then pressed under a heat press machine (Digital Combo 16, GeoKnight & Co, Inc., Brockton, MA, USA) at 160 °C for 10 s.

### 2.3. Layer-by-Layer Polyelectrolyte Deposition

The as-prepared nanofiber sheets were expected to be negatively-charged at the surface due to the additional layer of PAA nanofibers. A polyelectrolyte bilayer was then prepared on the surface of the nanofiber sheets by sequential immersion in positively-charged PEI and negatively-charged PAA solutions. The nanofiber sheet was immersed in 0.5 wt % PEI solution for 10 min, rinsed with DI water and dried using air knife. The sheet was then immersed in 0.5 wt % PAA solution for another 10 min, then rinsed with DI water. Multiple immersion cycles were performed as well.

### 2.4. Interfacial Polymerization

The selective polyamide layer was formed on the side wherein polyelectrolyte bilayer was formed prior. The nanofiber membrane support was first dried using a rubber roller, then immersed in 2 wt % MPD solution for 2 min. Excess MPD solution was removed from the surface using rubber roller, and the membrane surface was immersed in 0.15 wt % TMC solution for 1 min. The excess TMC solution was drained, and the membrane was air-dried for 2 min then oven-dried at 90 °C for 3 min. The prepared nanofiber-supported TFC membrane was then preserved in DI water until tested. A control TFC membrane, without polyelectrolyte deposition, was also prepared.

### 2.5. Osmotic Performance

Osmotic water flux and reverse salt flux of the TFC membranes were evaluated sing a custom lab-scale cross-flow FO system. NaCl concentrations of 0.5, 1.0, 1.5, and 2.0 M were used as draw solutions while deionized water (DI) was used as the feed solution. Osmotic flux tests were conducted in FO mode (i.e., the membrane active layer facing the feed solution) and PRO mode (i.e., the membrane active layer facing the draw solution) orientations. The hydraulic pressures of the feed and draw solutions were kept at minimum, and the cross-flow velocities and flow rates for both were kept at 0.014 m s^−1^ and 0.500 L min^−1^, respectively. The temperature of the feed and draw solutions were maintained at 25.0 ± 1 °C using a water bath. Membranes were pre-wetted in 50% IPA prior to water flux test for 30 s to saturate the porous structure of the membrane. An electronic top-loading balance (CP 2002, Ohaus Instrument Co., Ltd., Parsipanny, NJ, USA) connected to a computer recorded the mass of permeated water into the draw solution. Change in conductivity of the DI feed solution was measured to calculate reverse salt flux. FO was operated for at least 30 min to obtain stable measurements. All measurements were performed in triplicate.

Water flux (J_w_, L m^−2^ h^−1^) was calculated using Equation (1):

(1) $$J_{w}=\;\frac{\Delta m\;}{S_{m}\Delta {t\rho }_{w}}$$

where Δm, S_m_, and Δt, are change in mass of feed solution, effective membrane surface area, and change in time, respectively. Reverse salt flux (J_s_, g m^−2^ h^−1^), on the other hand, was calculated using Equation (2):

(2) $$\;J_{s}=\;\frac{\Delta \left(C_{t}V_{t}\;\right)}{S_{m}\Delta t}$$

where C_t_ and V_t_ are salt concentration and feed volume at time t, respectively. The specific salt flux is the ratio of reverse salt flux and water flux, J_s_/J_w_.

### 2.6. Determination of Membrane Parameters

Membrane parameters, pure water permeability (A) and solute permeability coefficient (B) were determined using a cross-flow reverse osmosis (RO) filtration system (Sterlitech Co., Kent, WA, USA), with an effective membrane area of 42 cm^2^. Prior to the flux test, the membranes were placed in DI at 5 bar for 1 h to eliminate possibility of membrane compaction.

Pure water flux through the membrane was measured at various transmembrane pressures (TMP) from 1 to 10 bar with a flow rate and cross-flow velocity of 1.5 L min^−1^ and 0.25 m s^−1^, respectively. A was calculated using Equations (3) and (4):

(3) $$\;J_{w}=\;\frac{\Delta V\;}{A_{m}\Delta t}$$

(4) $$\;A=\;\frac{J_{w}\;}{\Delta P}$$

where ΔV, A_m_, Δt, and ΔP are permeate volume, effective membrane area, sampling time, and applied pressure, respectively [16].

Salt rejection (R) and solute permeability coefficient were determined after performing a flux test for 1 h with 1000 mg L^−1^ NaCl solution as draw solution and the following system conditions: 25 °C and 10 bar. R and B are calculated using Equations (5) and (6):

(5) $$\;R=\;\left(1-\;\frac{C_{d}\;}{C_{f}}\right)$$

(6) $$\;B={\;J\;}_{w}\left(\frac{1-R}{R}\right)\;\exp\left(-\frac{J_{w}}{k}\right)$$

where C_f_, C_d_, and k are the solute concentrations of the feed and draw solutions and mass transfer coefficient, respectively [4]. k is a function of the solute diffusion coefficient (D), hydraulic diameter (d_h_) of the cross flow cell, and the Sherwood number (Sh), which is calculated based on the hydrodynamic conditions of the FO system, as shown in Equations (7)–(9):

(7) $$\;k=\;\frac{\mathrm{Sh}\;\cdot D\;}{d_{h}}$$

(8) Sh=1.85(Re·ScdhL)0.33     if Re < 2000

(9)  Sh=0.04 (Re 0.75·Sc0.33)     if Re > 2000

where Re, Sc, and L are Reynolds number, Schmidt number, and length of the channel, respectively [29,30].

The membrane structural parameter (S) was determined after performing an FO test on the membrane, and calculated using Equation (10):

(10)  S= KD 

where K is the solute resistance to diffusion within the membrane support layer [31,32]. 

### 2.7. Membrane Characterization

#### 2.7.1. Surface and Cross-Section Morphology

The surface and cross-section morphology of the PVDF/CTA membranes were examined under a field emission scanning electron microscope (FESEM, Zeiss SUPRA 55-VP, Oberkochen, Germany). Prior to FESEM analysis, the membrane samples were dried before sputter-coated with 10 nm of gold and palladium. For cross-section morphology analysis, the membrane samples were frozen using liquid nitrogen and snapped immediately prior to sputter-coating. 

#### 2.7.2. Water Contact Angle

The hydrophilicity of the membrane was measured using an optical tensiometer (Attension Theta, Biolin Scientific, Gothenberg, Sweden), employing the sessile drop method. A 5-µL water droplet was made to contact the membrane, and contact angle values were recorded through OneAttension software (Biolin Scientific). The average of five measurements on different spots of the membrane was reported.

#### 2.7.3. Pore Size and Porosity Determination

Membrane porosity was determined via gravimetric analysis [24]. Pre-weighed dried samples were soaked in water for 24 h at 30 °C, and the wet samples were re-weighed. Porosity (ε) was calculated through Equation (11):

(11) $$\;\varepsilon =\;\frac{\frac{\left(m_{2}-m_{1}\;\right)}{\rho_{w}}}{\frac{\left(m_{2}-{\;m}_{1}\right)}{\rho_{w}}+\;\frac{m_{2}}{\rho_{p}}}$$

where m_1_, m_2_, ρ_w_, and ρ_p_ are weight of the dry sample, weight of the wet sample, density of water, and density of the polymer, respectively.

#### 2.7.4. Membrane Mechanical Strength and Thickness

Mechanical strength of the membrane was determined using an advanced material testing system (Lloyd Materials Testing LS1, Ametek, Berwyn, PA, USA) with a 1 kN load cell. The membrane samples were cut into 30 mm × 10 mm prior to the test. Membrane thickness was determined using a digital micrometer (RS Pro Micrometer, RS Components, Sydney, NSW, Australia).

#### 2.7.5. Surface Chemistry Characterization

Fourier transform infrared (FTIR) spectroscopy (IRAffinity-1, Shimadzu, Kyoto, Japan) equipped with a single reflection attenuated total reflectance (ATR, MIRacle 10, Shimadzu, Kyoto, Japan) was used to analyze the chemical composition of the nanofiber supports after the layer-by-layer polyelectrolyte deposition.

## 3. Results and Discussion

### 3.1. Properties of Nanofiber PVDF Membrane Support

The nanofiber PVDF support of the TFC membrane was fabricated and coated with PAA using electrospinning technique. The melting point of PVDF is at the range of 165 to 172 °C, while that of PAA is 106 °C. The PVDF nanofibers were coated with PAA to obtain a negative surface charge, to make it more susceptible for LbL deposition of electrolytic solutions. The nanofibers were heat-treated at 160 °C, a temperature close to but not exceeding the melting point of PVDF, to enhance the mechanical strength of the fibers [24]. Heat press treatment is expected to allow conjugation of the nanofibers to occur.

**Figure 2:**
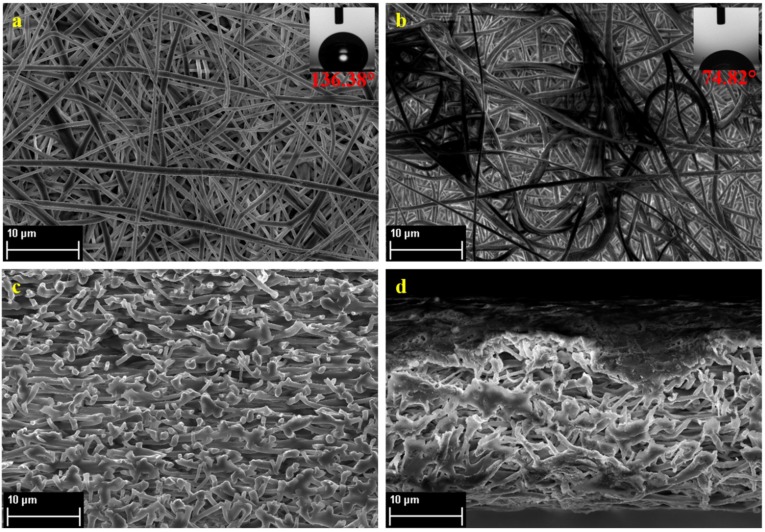
FESEM images of the (**a,b**) top surface of and (**c,d**) cross section of pure PVDF and PVDF-PAA nanofiber mats, respectively.

Figure 2 shows the FESEM images of the PVDF nanofiber mats of pure PVDF and PVDF-PAA. Figure 2a shows that the PVDF nanofibers have a bead-free structure, indicating a smooth and uniform fibrous surface of PVDF [23]. Figure 2b, on the other hand, shows that PAA was shown to have melted during the heat press treatment at 160 °C, forming a slight thin film on top of the PVDF nanofibers.

The average fiber diameter were 881 ± 294 and 934 ± 327 nm for PVDF and PVDF-PAA, respectively, showing closely similar values for both nanofiber membrane supports, indicating that the PAA nanofibers have melted during heat press treatment and produced a thin coating on the PVDF nanofibers. While the morphology and average fiber diameter of the two nanofiber supports revealed no significant differences, surface hydrophilicity of the nanofibers, as shown by contact angle measurements, changed drastically after electrolytic coating with PAA. Plain PVDF nanofibers exhibited a contact angle measurement of 136.38°, while after PAA coating, the contact angle dramatically decreased to 74.82°. It is well known that, a relatively high hydrophobicity of PVDF is due to its structure, as well as the low surface energy of PVDF [33]. The change in hydrophilicity observed indicates that PAA nanofibers were successfully spun onto the PVDF nanofibers. PAA is a chain of monomers containing a carboxylic acid –COOH group, which is known to be hydrophilic. This is also why PAA can be dissolved in an aqueous solution of 0.5 M NaCl. Since the dope solution was prepared with a mixture of NaCl, the PAA species is expected to exhibit a negative charge. Ensuring that PAA was successfully coated on the nanofiber mat also indicates that further immersion of the nanofibers in electrolytic solution may possible due to the presence of a charged species on the nanofiber surface.

Comparing the mechanical properties (tensile strength, elongation, and Young’s modulus) of the plain PVDF and PVDF-PAA nanofiber mats, shown in Table 1, it can be seen that the mechanical properties of PVDF nanofibers were significantly improved after coating with PAA. This is most likely due to the thicker deposition of nanofibers, after electrospinning of PAA for three additional hours and heat press treatment. Heat press treatment of the nanofibers resulted to better connectivity of the nanofibers, resulting to reinforced strength of the nanofiber mats. Furthermore, previous studies have also suggested that PAA can also act as an adhesive for various systems [34,35]. It is highly possible that heat press treatment of PAA nanofibers resulted to melting, facilitating further adhesion among the PVDF nanofibers. This would lead to the enhanced mechanical strength of the nanofiber membrane supports.

Porosity and water uptake capability of the nanofiber mats were also compared and shown in Table 1. While the porosity of the pure PVDF nanofibers and PVDF-PAA nanofibers were insignificant, the additional electrospinning of PAA onto the PVDF nanofiber mat has definitely enhanced the hydrophilic characteristic of the nanofiber support, as earlier shown by its surface contact angle, and its water uptake capacity of 138.21%, compared to 4.29% of plain PVDF. No significant changes in the mechanical properties and porosity were observed after the LbL treatment; these show that the LbL-treated PVDF nanofibers are similar in properties with the PVDF-PAA nanofibers.

### 3.2. Molecular Layer-by-Layer Approach

**Figure 3:**
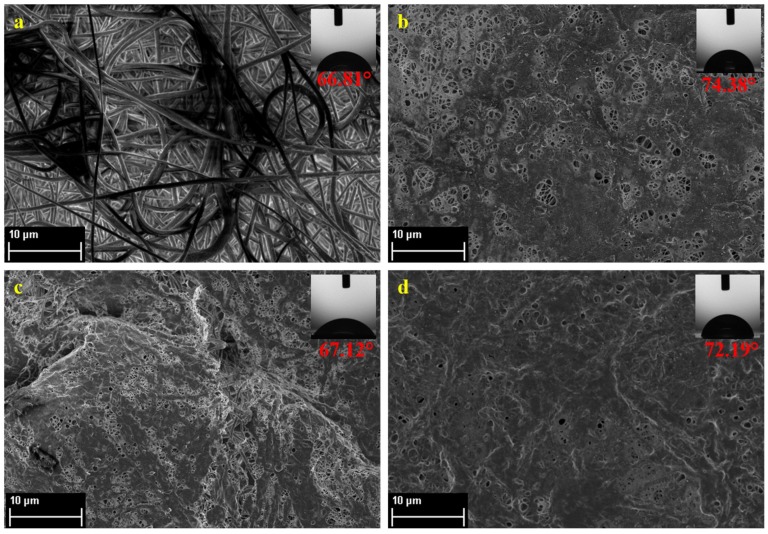
The surface morphology of the membranes shown by FESEM imaging for each electrolyte deposition: (**a**) initial PVDF-PAA nanofibers; (**b**) after immersion in PEI for 10 min; (**c**) after immersion in PEI and PAA for 10 min each; (**d**) after two cycles of PAA and PEI immersion for 10 min each.

Prior to IP, the PVDF-PAA nanofiber mats were subjected to LbL approach by immersion of the nanofiber mat surface in electrolytic solutions, 0.5 wt % PEI and 0.5 wt % PAA, both in 0.5 M NaOH, which carry the positive and negative charges, respectively. Figure 3a shows the PVDF-PAA nanofiber prior to LbL. Due to the negative charge of PAA, the nanofiber mat was first immersed in positively-charged PEI to form a neutrally-charged layer. The reaction of the carboxylic acid groups of PAA and the amino groups of PEI react together electrostatically and due to the presence of H-bonds [36]. Figure 3b shows the first layer of the PAA and PEI. The contact angle of the nanofiber mat after the immersion with PEI increased to 74.38°, indicating the slightly hydrophobic character of polyamide. Based on Figure 3b, the surface of the nanofiber still exhibited the presence of pores and non-uniform coating, thus another deposition cycle of both PAA and PEI was performed, resulting to the nanofiber mats whose morphologies are shown in Figure 3c,d. After two deposition cycles of PAA and PEI, formation of two polyelectrolyte layers ensures a more uniform coating on the nanofiber support, which was then proceeded for the IP process to form the polyamide selective layer. The final PVDF-LbL support exhibited a final surface contact angle of 72.19°, indicating highly satisfactory hydrophilicity.

**Figure 4:**
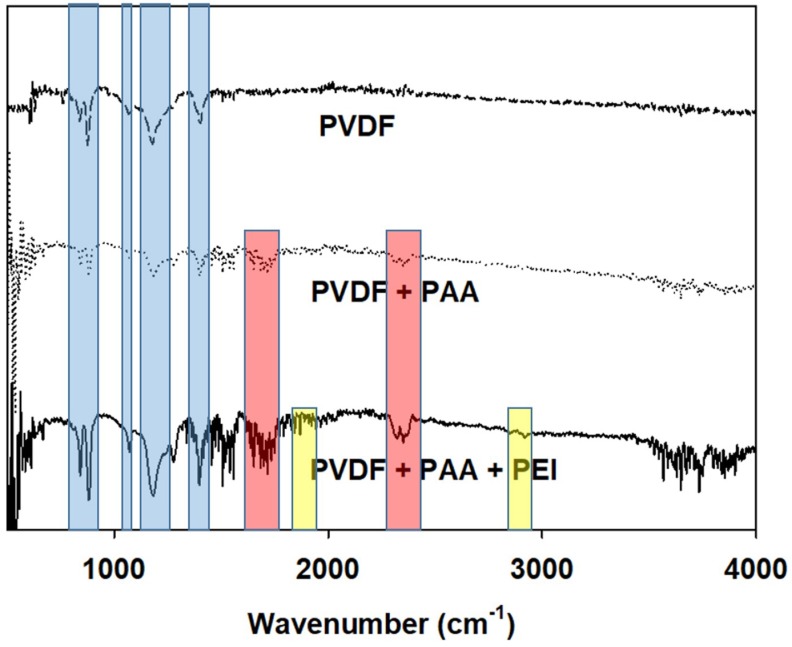
The FTIR spectra of the plain PVDF nanofibers, PVDF/PAA nanofibers, and PVDF/PAA nanofiber with the polyelectrolyte mat with PAA and PEI deposition.

The chemical composition of the plain PVDF nanofiber mat and its subsequent modifications was characterized using FTIR, and the spectra were shown in Figure 4. The plain PVDF nanofiber mat showed the typical peaks for PVDF polymer (1400 cm^−1^ for the C–H stretching vibration, and 840 cm^−1^, 1180 cm^−1^, and 1275 cm^−1^, which are all representative of the C–F bonds present in PVDF) [37,38]. These peaks are likewise present in the modified samples, indicating that PVDF remains an integral part of the support layer, despite numerous modifications. However, for the nanofiber mat containing both PVDF and PAA nanofibers, peaks at 1700 cm^−1^ and 2350 cm^−1^ were found, characteristic of the COOH and C=O bonds, respectively of PAA. Furthermore, weak peaks are also found at 1350 and 1500 cm^−1^, corresponding to the COO– group. These peaks are also found in the nanofiber mat modified by LbL deposition of PAA and PEI. The modified PVDF-LbL nanofiber support has shown peaks at 1750 cm^−1^ and 2930 cm^−1^, which correspond to N–H and CH_2_, respectively, which are both characteristic of PEI.

### 3.3. Properties of the TFC Membranes

Polyamide selective layers were deposited on both plain PVDF and modified PVDF-LbL nanofiber supports via IP reaction of MPD and TMC.

**Figure 5:**
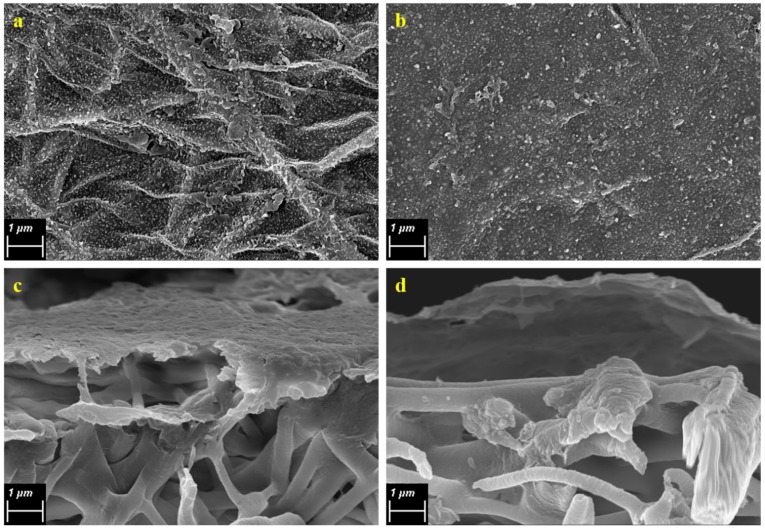
The (**a,b**) surface morphology and (**c,d**) cross-section morphology of the control TFC and the PVDF-LbL TFC membrane, respectively.

FESEM images (Figure 5a,b) show the surface morphology of the PVDF TFC and PVDF-LbL TFC membranes. Typical ridge-and-valley structures of polyamide were shown by both the membrane samples, indicating that polyamide was formed well onto the nanofiber mat. The difference observed for the samples, however, is that, for PVDF TFC membrane, the structure of the nanofiber surface was clearly visible beneath the polyamide layer of the PVDF TFC membrane, which was not observed with the PVDF-LbL TFC membrane. This shows that the uniform coating and deposition of PAA and PEI layers have occurred for the latter. Cross-section images (Figure 5c,d) of the TFC membranes show that for PVDF TFC membrane, the polyamide layer is directly on top of the nanofibers, while two layers can be seen on top of the nanofibers of PVDF-LbL TFC membrane, corresponding to the polyelectrolyte and polyamide layers.

Upon determination of the contact angle of both membranes, it was observed that the polyamide layer had similar hydrophilic character to the polyelectrolyte layer formed from the reaction of PAA and PEI. PVDF and PVDF-LbL TFC membranes showed contact angles of 94.18° and 92.21°, respectively. This indicated that, while the TFC membranes are not as hydrophilic as the PVDF-PAA nanofibers, the resultant TFC membranes were still more hydrophilic than the PVDF nanofibers.

### 3.4. Membrane Performance

The TFC membranes were tested for forward osmosis and their performance were compared to that of the commercial CTA FO membrane from HTI. The membranes underwent FO operation at two different membrane orientations, or modes: FO mode, wherein the active layer faces the feed solution (AL-FS), and PRO mode, wherein the active layer faces the draw solution (AL-DS). DI water was used as the feed, while various concentrations of NaCl were used as the draw.

**Figure 6:**
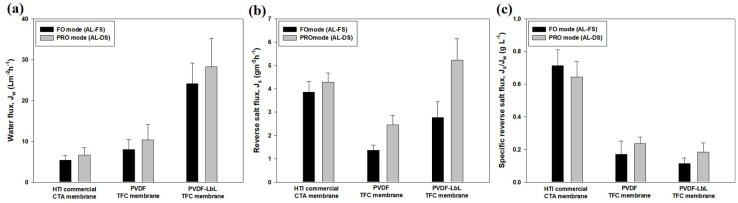
Membrane performance for FO operation of the (**a**) PVDF TFC membrane; (**b**) PVDF-LbL TFC membrane; and (**c**) commercial CTA membrane at FO (AL-FS, i.e., active layer facing the feed solution) and PRO (AL-DS, i.e., active layer facing the draw solution) modes with 0.5 M NaCl and DI water as the draw and feed solutions, respectively.

Figure 6 shows the performance of the PVDF TFC and PVDF-LbL TFC membranes during FO operation at FO and PRO modes, and compared with the performance of the commercial CTA membrane with with 0.5 M NaCl and DI water as the draw and feed solutions, respectively. Among the three membranes tested, the PVDF-LbL membrane exhibited the highest water flux values of 24.1 and 28.3 L m^−2^ h^−1^ for FO and PRO mode, respectively, followed by the PVDF TFC membrane with 8.0 (FO mode) and 10.4 (PRO mode) L m^−2^ h^−1^. The commercial CTA membrane with water fluxes of 5.4 (FO mode) and 6.7 (PRO mode) L m^−2^ h^−1^ was the lowest-performing compared to the other two membranes. Besides, the commercial CTA membrane showed the lowest water flux values, it also exhibited the highest specific reverse salt flux (ratio of J_s_/J_w_) values of 0.643 to 0.714 g L^−1^, compared to the TFC membranes (0.115 to 0.236 g L^−1^).

It is noticeable that the membranes all exhibited lower water flux and reverse salt fluxes under FO mode compared to PRO mode of operations and this phenomenon is a result of the dilutive ICP within the membrane support layer, which significantly reduces the osmotic pressure difference during FO mode [24]. Although the PRO mode of operation results in higher water fluxes, it also enhances the reverse solution permeation. As a result, the specific reverse salt flux (ratio of J_s_/J_w_) values under both the operation modes were found to be not significantly different. 

**Figure 7:**
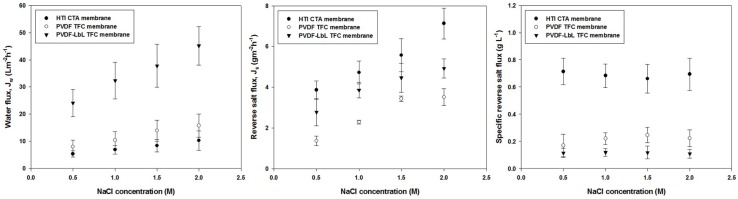
Membrane performance for FO operation of the PVDF TFC membrane, PVDF-LbL TFC membrane and commercial CTA membrane at different concentrations of NaCl (0.5–2.0 M) as draw solution and DI water as feed solution.

Figure 7 shows the membrane performance of the TFC and commercial CTA membranes at various draw solution concentrations of 0.5 to 2.0 M NaCl. It can be observed that both water fluxes and reverse salt fluxes of the membranes increased at a higher draw solution concentrations, which is expected because of the higher osmotic pressure driving force of the draw solutions [39]. Both the water and reverse salt fluxes of the membranes increased at higher draw solute concentrations; however, the specific reverse salt fluxes (ratio of J_s_/J_w_) of the particular membranes remained similar all throughout the FO operation, irrespective of the draw solution concentration used. It is also noteworthy that the J_s_/J_w_ values of both the TFC membranes are not significantly different despite significant differences in the water and reverse solute fluxes, and this is likely due to the presence of polyamide layer, which have similar rejecting properties for both the membranes. A TFC membrane with polyamide active layer is generally reported to have higher water permeability and lower solute permeability compared to CTA membranes [40].

Table 2 shows the membrane intrinsic transport parameters of the three FO membranes. The pure water permeability coefficient (A value) of the PVDF TFC membrane was 1.88 L m^−2^ h^−1^ bar^−1^, which significantly increased to 4.12 L m^−2^ h^−1^ bar^−1^ for the PVDF-LbL TFC membrane, consistent with the earlier characterization and performance tests. Compared with the two TFC membranes, the commercial CTA membrane exhibited lower A values of 0.64 L m^−2^ h^−1^ bar^−1^, indicating poorer water permeability. The commercial CTA membrane also exhibited low A values, it showed the highest solute permeability coefficient (B value of 0.57 L m^−2^ h^−1^) among the three samples tested, while the two TFC membranes showed similar B values both lower than that of the CTA membrane.

The membrane structural parameter (S) is one of the indicators of the occurrence of ICP that significantly affects FO membrane performance. The membrane with higher S values tend to exhibit higher ICP compared to membranes with lower S values. As expected, the PVDF-LbL TFC membrane showed the lowest S value of 221 µm, compared to the commercial CTA membrane (721 µm) and the PVDF TFC membrane (482 µm).

Table 3 shows a performance comparison of the PVDF-LbL TFC membranes in this study with other nanofiber-supported TFC membranes for FO application. The comparison table shows that the performance of our PVDF-LbL TFC membranes were comparable with those in literature, despite the ease in preparation and practicality of modification approach.

## 4. Conclusions

The molecular layer-by-layer modification was successfully performed to significantly enhance the properties and the performance of nanofiber PVDF-supported TFC membranes. Electrospun PVDF nanofibers were initially coated with PAA, a negatively-charged electrolytic polymer, via electrospinning. The resultant nanofiber sheet underwent heat press treatment, prior to layer-by-layer deposition of PEI and PAA, to form a polyelectrolyte layer, whose structure is highly similar to that of polyamide. After the polyelectrolyte layer deposition, interfacial polymerization was performed to form the selective polyamide layer and obtain improved performance of nanofiber-supported TFC membrane in terms of water permeability and structural parameter. This study observed that the layer-by-layer deposition of polyelectrolyte is a feasible modification method for improvement of hydrophilic property, as well as formation of polyamide active layer, of a nanofiber-supported TFC membrane.

## Figures and Tables

**Table 1:** Mechanical properties and porosity of the plain PVDF, PVDF-PAA, and PVDF-LbL nanofiber supports.

Property	PVDF Nanofiber	PVDF-PAA Nanofiber	PVDF-Lbl
Tensile Strength (MPa)	7.14 ± 0.61	8.51 ± 0.38	8.89 ± 0.71
Elongation (%)	138.12 ± 37.88	177.21 ± 18.03	191.16 ± 15.34
Young’s Modulus (MPa)	36.19 ± 11.14	68.31 ± 6.18	72.15 ± 10.14
Porosity (%)	79.21 ± 1.37	71.05 ± 0.68	72.18 ± 1.19
Water Uptake (%)	4.29 ± 0.45	138.21 ± 8.41	127.18 ± 5.88

**Table 2:** The membrane intrinsic transport properties of the PVDF TFC, PVDF-LbL TFC, and commercial CTA membrane.

Membrane	A (L m^−2^ h^−1^ bar^−1^)	B (L m^−2^ h^−1^)	R (%)	S (µm)
HTI CTA	0.64	0.57	92.18	721
PVDF TFC	1.88	0.43	95.17	482
PVDF-LbL TFC	4.12	0.38	96.43	221

**Table 3:** Performance comparison of the nanofiber-supported TFC FO membranes in literature with the PVDF-LbL TFC membrane.

Membrane	Draw Solution	J_w_ (L m^−2^ h^−1^)	J_s_ (g m^−2^ h^−1^)	J_s_/J_w_ (g L^−1^)	Reference
Nylon 6,6-modified PVDF	0.5 M NaCl	16.0	2.7	0.17	[5]
PVA ^a^	0.5 M NaCl	27.7	-	-	[21]
PET ^b^-supported CA ^c^/PAN ^d^	1.5 M NaCl	27.6	3.9	0.14	[22]
PVDF-PVA	0.5 M NaCl	24.8	3.3	0.13	[24]
PET/PVA (1:4)	0.5 M NaCl	47.2	9.5	0.20	[26]
Nylon 6,6	1.0 M NaCl	21.0	5.2	0.24	[41]
TEA ^e^-modified PVDF	2.0 M NaCl	68.0	2.0	0.03	[42]
PVDF	0.5 M NaCl	18.5	2.7	0.14	[43]
PVDF/CA composite	0.5 M NaCl	20.2	2.1	0.10	
PVDF/CA blend	0.5 M NaCl	31.3	0.8	0.03	
PVDF	1.0 M NaCl	28.0	12.9	0.46	[44]
PVDF-LbL	0.5 M NaCl	24.1	2.8	0.12	This work
	1.0 M NaCl	32.4	3.9	0.12	
	1.5 M NaCl	37.8	4.5	0.12	
	2.0 M NaCl	45.2	4.9	0.11	

^a^ Polyvinyl alcohol; ^b^ Polyethylene terepththalate; ^c^ Cellulose acetate; ^d^ Polyacrylonitrile; ^e^ Triethylamine.

## References

[1] Cath T.Y., Childress A.E., Elimelech M. (2006). Forward osmosis: Principles, applications, and recent developments. J. Membr. Sci..

[2] Cutcheon M.J.R., Elimelech M. (2006). Influence of concentrative and dilutive internal concentration polarization on flux behavior in forward osmosis. J. Membr. Sci..

[3] Yip N.Y., Tiraferri A., Phillip W.A., Schiffman J.D., Elimelech M. (2010). High performance thin-film composite forward osmosis membrane. Environ. Sci. Technol..

[4] Tiraferri A., Yip N.Y., Phillip W.A., Schiffman J.D., Elimelech M. (2011). Relating performance of thin-film composite forward osmosis membranes to support layer formation and structure. J. Membr. Sci..

[5] Huang L., Arena J.T., Cutcheon M.J.R. (2016). Surface modified PVDF nanofiber supported thin film composite membranes for forward osmosis. J. Membr. Sci..

[6] Alsvik I.L., Hägg M.B. (2013). Pressure retarded osmosis and forward osmosis membranes: Materials and methods. Polymers.

[7] Lee K.L., Baker R.W., Lonsdale H.K. (1981). Membranes for power generation by pressure-retarded osmosis. J. Membr. Sci..

[8] Cadotte J.E., Petersen R.J., Larson R.E., Erickson E.E. (1980). A new thin-film composite seawater reverse osmosis membrane. Desalination.

[9] Peyki A., Rahimpour A., Jahanshahi M. (2015). Preparation and characterization of thin film composite reverse osmosis membranes incorporated with hydrophilic SiO_2_ nanoparticles. Desalination.

[10] Wei J., Qiu C., Tang C.Y., Wang R., Fane A.G. (2011). Synthesis and characterization of flat-sheet thin film composite forward osmosis membranes. J. Membr. Sci..

[11] Klaysom C., Hermans S., Gahlaut A., Van Craenenbroeck S., Vankelecom I.F.J. (2013). Polyamide/Polyacrylonitrile (PA/PAN) thin film composite osmosis membranes: Film optimization, characterization and performance evaluation. J. Membr. Sci..

[12] Chung T.S., Li X., Ong R.C., Ge Q., Wang H., Han G. (2012). Emerging forward osmosis (FO) technologies and challenges ahead for clean water and clean energy applications. Curr. Opin. Chem. Eng..

[13] Han G., Zhang S., Li X., Chung T.S. (2015). Progress in pressure retarded osmosis (PRO) membranes for osmotic power generation. Prog. Polym. Sci..

[14] Li G., Li X.M., He T., Jiang B., Gao C. (2013). Cellulose triacetate forward osmosis membranes: Preparation and characterization. Desalination Water Treat..

[15] Cui Y., Liu X.Y., Chung T.S. (2014). Enhanced osmotic energy generation from salinity gradients by modifying thin film composite membranes. Chem. Eng. J..

[16] Park M.J., Phuntsho S., He T., Nisola G.M., Tijing L.D., Li X.M., Chen G., Chung W.J., Shon H.K. (2015). Graphene oxide incorporated polysulfone substrate for the fabrication of flat-sheet thin-film composite forward osmosis membranes. J. Membr. Sci..

[17] Choi W., Jeon S., Kwon S.J., Park H., Park Y.I., Nam S.E., Lee P.S., Lee J.S., Choi J., Hong S. (2017). Thin film composite reverse osmosis membranes prepared via layered interfacial polymerization. J. Membr. Sci..

[18] Song X., Liu Z., Sun D.D. (2013). Energy recovery from concentrated seawater brine by thin-film nanofiber composite pressure retarded osmosis membranes with high power density. Energy Environ. Sci..

[19] Bui N.N., Lind M.L., Hoek E.M.V., Cutcheon M.J.R. (2011). Electrospun nanofiber supported thin film composite membranes for engineered osmosis. J. Membr. Sci..

[20] Song X., Liu Z., Sun D.D. (2011). Nano gives the answer: Breaking the bottleneck of internal concentration polarization with a nanofiber composite forward osmosis membrane for a higher water production rate. Adv. Mater..

[21] Puguan J.M.C., Kim H.S., Lee K.J., Kim H. (2014). Low internal concentration polarization in forward osmosis membranes with hydrophilic crosslinked PVA nanofibers as porous support layer. Desalination.

[22] Bui N.N., Cutcheon M.J.R. (2013). Hydrophilic nanofibers as new supports for thin film composite membranes for engineered osmosis. Environ. Sci. Technol..

[23] Bui N.N., Cutcheon M.J.R. (2014). Nanofiber supported thin-film composite membrane for pressure-retarded osmosis. Environ. Sci. Technol..

[24] Park M.J., Gonzales R.R., Wahab A.A., Phuntsho S., Shon H.K. (2018). Hydrophilic polyvinyl alcohol coating on hydrophobic electrospun nanofiber membrane for high performance thin film composite forward osmosis membrane. Desalination.

[25] Tijing L.D., Woo Y.C., Johir M.A.H., Choi J.S., Shon H.K. (2014). A novel dual-layer bicomponent electrospun nanofibrous membrane for desalination by direct contact membrane distillation. Chem. Eng. J..

[26] Tian E.L., Zhou H., Ren Y.W., Mirza Z., Wang X.Z., Xiong S.W. (2014). Novel design of hydrophobic/hydrophilic interpenetrating network composite nanofibers for the support layer of forward osmosis membrane. Desalination.

[27] Huang L., Manickam S.S., Cutcheon M.J.R. (2013). Increasing strength of electrospun nanofiber membranes for water filtration using solvent vapor. J. Membr. Sci..

[28] Huang L., Cutcheon M.J.R. (2014). Hydrophilic nylon 6,6 nanofibers supported thin film composite membranes for engineered osmosis. J. Membr. Sci..

[29] Tan C.H., Ng H.Y. (2008). Modified models to predict flux behavior in forward osmosis in consideration of external and internal concentration polarizations. J. Membr. Sci..

[30] Tan C.H., Ng H.Y. (2013). Revised external and internal concentration polarization models to improve flux prediction in forward osmosis process. Desalination.

[31] Gerstandt K., Peinemann K.V., Skilhagen S.E., Thorsen T., Holt T. (2008). Membrane processes in energy supply for an osmotic power plant. Desalination.

[32] Li X.M., He T., Dou P., Zhao S. (2010). Forward Osmosis and Forward Osmosis Membranes. Reference Module in Chemistry, Molecular Sciences and Chemical Engineering.

[33] Pan Y., Wang W., Peng C., Shi K., Luo Y., Ji X. (2014). Novel hydrophobic polyvinyl alcohol-formaldehyde foams for organic solvents absorption and effective separation. RSC Adv..

[34] Onuki Y., Nishikawa M., Morishita M., Takayama K. (2008). Development of photocrosslinked polyacrylic acid hydrogel as an adhesive for dermatological patches: Involvement of formulation factors in physical properties and pharmacological effects. Int. J. Pharm..

[35] Sugama T., Kukacka L.E., Clayton C.R., Hua H.C. (1987). Effects of polyacrylic acid primers on adhesion and durability of FPL-etched aluminum/polyurethane systems. J Adhes. Sci. Technol..

[36] Choi W., Gu J.E., Park S.H., Kim S., Bang J., Baek K.Y., Park B., Lee J.S., Chan E.P., Lee J.H. (2015). Tailor-made polyamide membranes for water desalination. ACS Nano.

[37] Obaid M., Ghouri Z.K., Fadali O.A., Khalil K.A., Almajid A.A., Barakat N.A. (2016). Amorphous SiO_2_ NP-Incorporated Poly(vinylidene fluoride) Electrospun Nanofiber Membrane for High Flux Forward Osmosis Desalination. ACS Appl. Mater. Interfaces.

[38] Zeng Z., Yu D., He Z., Liu J., Xiao F.X., Zhang Y., Wang R., Bhattacharyya D., Tan T.T. (2016). Graphene Oxide Quantum Dots Covalently Functionalized PVDF Membrane with Significantly-Enhanced Bactericidal and Antibiofouling Performances. Sci. Rep..

[39] Han G., Cheng Z.L., Chung T.S. (2017). Thin-film composite (TFC) hollow fiber membrane with double-polyamide active layers for internal concentration polarization and fouling mitigation in osmotic processes. J. Membr. Sci..

[40] Ren J., Cutcheon M.J.R. (2014). A new commercial thin film composite membrane for forward osmosis. Desalination.

[41] Huang L., Bui N.N., Meyering M.T., Hamlin T.J., Cutcheon M.J.R. (2013). Novel hydrophilic nylon 6,6 microfiltration membrane supported thin film composite membranes for engineered osmosis. J. Membr. Sci..

[42] Obaid M., Mohamed H.O., Yasin A.S., Fadali O.A., Khalil K.A., Kim T., Barakat N.A.M. (2016). A novel strategy for enhancing the electrospun PVDF support layer of thin-film composite forward osmosis membranes. RSC Adv..

[43] Shibuya M., Park M.J., Lim S., Phuntsho S., Matsuyama H., Shon H.K. (2018). Novel CA/PVDF nanofiber supports strategically designed via coaxial electrospinning for high performance thin-film composite forward osmosis membranes for desalination. Desalination.

[44] Tian M., Qiu C., Liao Y., Chou S., Wang R. (2013). Preparation of polyamide thin film composite forward osmosis membranes using electrospun polyvinylidene fluoride (PVDF) nanofibers as substrates. Sep. Purif. Technol..

